# A Groupware Tool to Facilitate Caregiving for Home-Dwelling Frail Older Persons in the Netherlands: Mixed-Methods Study

**DOI:** 10.2196/10697

**Published:** 2018-12-07

**Authors:** Hanneke Breebaart, Marjolein Broese van Groenou

**Affiliations:** 1 Department of Sociology Faculty of Social Sciences Vrije Universiteit Amsterdam Netherlands

**Keywords:** devices, kinship networks, long-term care, home care organization, communication, health, elderly, digital care network, digital networked communication

## Abstract

**Background:**

Collaboration among informal and formal caregivers in a mixed care network of home-dwelling elderly may benefit from using a groupware app for digital networked communication (DNC).

**Objective:**

This study aimed to describe and explain differences in the use and evaluation of a DNC app by members of the care network and to come up with a list of conditions that facilitate (or restrict) the implementation of a DNC app by a home care organization.

**Methods:**

A pilot study collected information on digital communication in 7 care networks of clients of a home care organization in the Netherlands. Semistructured interviews with 4 care recipients, 7 informal carers (of which 3 spoke on behalf of the care receiver as well on account of receivers’ suffering from dementia), 3 district nurses, 5 auxiliary nurses, and 3 managers were conducted 3 times in a period of 6 months. In addition, we observed relevant workshops initiated by the home care organization and studied log-in data created by the users of the DNC app.

**Results:**

The qualitative data and the monthly retrieved quantitative log-in data revealed 3 types of digital care networks: arranging the care network, discuss the care network, and staying connected network. Differences between network types were attributed to health impairment and digital illiteracy of the care recipients, motivation of informal caregivers, and commitment of formal caregivers. The easy availability of up-to-date information, the ability to promote a sense of safety for the carers, and short communication lines in case of complex care situations were positively evaluated.

**Conclusions:**

It is concluded that digital communication is beneficial for organizing and discussing the care within a care network. More research is needed to study its impact on care burden of informal carers, on quality of care, and on quality of life of home-dwelling frail older adults.

## Introduction

### Background

Frail community-dwelling older adults often receive care from formal and informal caregivers over a long period of time [1]. The presence of multiple types of caregivers and complex care tasks requires adequate communication on care to optimize its quality. However, communication between formal and informal caregivers is generally low, particularly in care networks lacking a cohabiting informal caregiver [2]. Moreover, when the care recipient is in poor health and less able to manage his or her own care, the low frequency of communication among formal caregivers and noncohabiting informal caregivers becomes a pressing issue for coordination tasks.

In the era of computer-supported cooperative work, the deployment of Web-based communication tools seems a logical way to organize care more efficiently around people at home [3]. Therefore, an increasing number of home care agencies are using a groupware tool to enable care coordination [4]. This type of software combines several functionalities such as registering goals and action plans, calendar managing, and networked communication. The empirical evidence regarding the effect of these apps on communication among different types of caregivers is limited. Most studies are only dealing with theoretical models of technology acceptance [5,6]. Moreover, a lot of studies are performed from a one-sided perspective only, such as the viewpoint of the care receiver [7]. Other computer-mediated health studies are limited to the communication in hospital care, for example, between the medical specialist and his patient at home [8].

To increase insight into how digital communication may improve communication between formal and informal caregivers in home settings, we conducted a pilot study among members of care networks around 7 clients of a home care organization in the Netherlands. The care recipients and their formal and informal caregivers were hooked onto an app developed for digital communication in a closed care network. We examined the use of the electronic tool (e-tool) for a period of 6 months using multiple ways of data collection. On the basis of acquired data, we aimed to (1) describe and explain differences in the use of the communication app by the members of the care network and (2) come up with a list of conditions that facilitate (or restrict) the implementation of such an app by a home care organization. The section below provides a short literature overview to identify conditions that predict differences in the actual use of the communication app and its effects on the process of caregiving.

### Online Care Networks

Crucial to all digital systems is the notion of networks. This is probably the most important distinguishing aspect of communication tools in comparison with other types of software [9]. Networked communication may help mixed home care networks to create online communities for coordinating care tasks and exchanging information. Similar to offline care networks, online care networks can be described according to their structural (size and composition) and functional characteristics (tasks, frequency, and content of discussion about care) [2]. In general, 3 types of offline mixed care networks are to be found among community-dwelling older care recipients: a small partner care network with few other helpers; the larger informal care network, composed of adult children, other relatives, nonkin, and formal caregivers; and the larger formal care network with few informal caregivers (mainly spouse or children) [10]. The type of care network present depends on the care needs and the economic, social, and psychological resources of the care recipient (eg, health status, income, partner status, and sense of self-efficacy). It can be expected that the composition of an online care network reflects the composition of an offline care network, but there are 2 important arguments that nuance this expectation.

First, not everybody desires or is able to use online tools. There are differences in determinants such as age, the severity of an illness, and attitudes toward digital networked communication (DNC) [11]. A questionnaire study based on the constructs of the unified theory of acceptance and use of technology showed that physical frailty status is associated with older peoples’ use of online tools, independent of age, education, and opinions on information and communications technology use [12]. Studies on care networks in which elderly were included showed that for people with mild dementia, technical errors and the unclearness of benefit lowered the initial trust in the groupware tool [13,14]. Such distrust in groupware tools may also be present among informal caregivers and home care professionals who are not common with these types of commercial tools. Another reason to refrain from DNC is that some highly valued characteristics of personal communication, for example, emotional support, do not translate into digital practices [15].

Second, offline communication between informal and formal caregivers largely depends on the meeting opportunities of the caregivers, defined by, for example, overlapping types of care activities and the coresidence of the informal caregiver [16]. In contrast, online communication provides ongoing meeting opportunities because any message communicated by any caregiver can be read by all other caregivers. However, assuming that for care recipients and informal caregivers there is no time limit to communication, this is limited for formal caregivers by their working hours and by how their organization has equipped teams to provide 24/7 attention to the needs of their clients [17]. In this line of reasoning, it is likely expected that an online care network represents only a part of the offline care network.

This leads to 2 main research questions (RQs) that guided our pilot study were:

RQ1: What are the characteristics of digital networked communication in terms of size and composition of the digital network and the frequency and content of communication?

RQ2: What might be the effects of using Web-based communication tools for the online and offline communication on care, the efforts from informal carers to help, and the perceived quality of care?

### The Role of the Home Care Organization and Usability of the Communication App

In addition to individual variation within the care network, there are several barriers and facilitators of success when implementing electronic health (eHealth) into care organizations. The extent to which the intervention fitted with the existing workflow and how well it is integrated within current working processes were found to influence implementation [18]. Nowadays, nurses experience a pressing responsibility of the economic aspect of their work; every task has to be done as efficiently as possible [19]. In addition, it is increasingly expected that they integrate informal caregiver involvement in formal work processes. Results of a study of 2 agencies and their clients in the Netherlands [20] show that the nurses were aware of the organizational policy which stated that they should proactively keep connected with informal caregivers, yet most of them acknowledged that they hardly ever did so. The main reasons were lack of time, too little initiative from the informal carer, and no clarity on which team member is responsible and accountable for the informal caregiver involvement. An app for DNC may deal with some of these issues and can be assistive for formal caregivers in stimulating informal helpers to communicate within online care networks. Currently, those groupware tools used in home care organizations are more comparable with Facebook, WhatsApp, and Google Calendar than with eHealth record software. The apps look attractive and are comfortable to use, and swiping to activate these types of digital tools is considered to be pleasant [21]. As they are installed on vehicles as mobile phones, all the members of the care network can communicate with each other synchronous and/or asynchronous while being in the same or different place [22]. Furthermore, using these apps instead of visiting someone saves time, and in contrast to telephone use, the information can be reread [4]. The above elaboration leads to the third research question:

RQ3: Which organizational conditions facilitate or restrict the implementation of a communication app in a home care organization?

## Methods

### Sample and Design

The research team, the app developer and a home care organization in a rural area in the eastern part of the Netherlands, agreed to arrange a pilot study to monitor and examine among clients of one specific team the implementation of the DNC app.

The app was software developed for digital communication in a closed network. Using an internet browser, the software could be implemented on an iPad, iPhone, and/or personal computer. It contained a calendar to schedule meetings and tasks, and users could leave electronic messages (e-messages) and photos. Users received prompts to indicate that new messages were present. The district nurses and team manager asked clients and/or their informal carers to participate in the pilot, and finally, 7 care networks around clients agreed to use the app. These networks all met the criterion that at least one informal caregiver and one formal home caregiver could be hooked onto the app. Other criteria for selection were not used, and clients varied in health conditions and living arrangements. Out of the 7 care receivers, 3 suffered from dementia to the extent that they could not participate themselves in interviews. In their case, informal caregivers spoke on their behalf. The 7 care networks started to use the app in February 2015 (T0). At the same time, information was collected on characteristics of the care recipient, the informal caregivers, and formal caregivers involved. The interviews were held face-to-face, by phone, or via Skype. Follow-ups were planned after 3 months (T1) and 6 months (T2). Monthly updates on log-in information were obtained from the developer of the tool. Auxiliary nurses who became members of the digital networks were interviewed during a focus group session, and field notes were made while observing 2 workshops to inform clients and/or their care network about the groupware tool. For the sake of completeness, 3 managers of the care agency were questioned about their vision and expectations on the DNC as part of the structural work of their staff. During the period of research, we conducted 42 semistructured interviews with 22 participants, consisting of 4 clients, 7 informal carers (of which 3 spoke on behalf of the care receiver as well), 3 district nurses, 5 auxiliary nurses, and 3 managers. Informed consents are obtained from the participants, and in field notes and transcriptions, their names are withheld for reasons of confidentiality.

### Procedure

Completed questionnaires were used to provide a short description of the clients and what type of care he or she got from whom. The gathered information was calculated using IBM SPSS version 23.0. Log-in data were provided monthly in an Excel sheet: actions, appointments, actors, and messages were coded (eg, action=entering text, appointment=going to the dentist, actor=district nurse, and message=care related/not care related), and the total number of different types of log-ins were counted. The audiotaped interviews were transcribed verbatim. For answering the 3 research questions, we investigated the transcriptions, field notes, and e-messages by using qualitative, directed content analysis [23]. The analysis consisted of reading and rereading the different types of data and writing down citations addressing the research questions. The next step was to search for linking themes and interpret them by means of constant comparison. Finally, the transcriptions are worked through by the qualitative research software Atlas.ti 7.5. Multiple ways of data collection have thus been used with multiple types of respondents. An overview of which information is provided by whom is given in Table 1.

**Table 1:** Overview of the data collection for answering the research questions.

Network, respondents, log data		T0		T1		T2	
**NW^a^1**							
	CL^b^		2^c^, 5^d^		3^e^		3, 5
	IC^f^		2, 5		3		3, 5
	Log data		Yes		Yes		Yes
**NW2**							
	IC on behalf of CL		2, 5		3		3, 5
	IC		2, 5		3		3, 5
	AN^g^		1^h^		1		N/A^i^
	Log data		Yes		Yes		Yes
**NW3**							
	IC on behalf of CL		2, 5		1, 3		3, 5
	IC		2, 5		3		3, 5
	AN		1		1		3
	Log data		Yes		Yes		Yes
**NW4**							
	CL		2, 5		1, 3		3, 5
	IC		3, 5		3		3, 5
	AN		1		1		4^j^
	Log data		Yes		Yes		Yes
**NW5**							
	IC on behalf of CL		2, 5		3		3, 5
	IC		2, 5		3		3, 5
	AN		1		1		3, 4
	Log data		Yes		Yes		Yes
**NW6**							
	CL		1, 5		2		3, 5
	IC		1, 5		2		3, 5
	DN^k^		1, 4, 5		1, 3		3, 5
	AN		N/A		1		4
	Log data		Yes		Yes		Yes
**NW7**							
	CL		N/A		1, 3, 5		3, 5
	IC		N/A		1, 3, 5		3, 5
	DN		N/A		3, 5		3, 5
	Log data		N/A		Yes		Yes
**NW1-5**							
	DN		1, 4, 5		1, 3, 5		3, 5
	DN of CL not started		N/A		3		N/A
	AN of CL not started		3		N/A		4
	Team manager		4		1		N/A
	Division manager		3		N/A		N/A
	Sector manager		3		N/A		N/A

^a^NW: network.

^b^CL: client.

^c^2: face-to-face interview.

^d^5: questionnaires.

^e^3: telephone or Skype interview.

^f^IC: informal carer.

^g^AN: auxiliary nurse.

^h^1: observation.

^i^N/A: not applicable.

^j^4: focus group interview.

^k^DN: district nurse.

## Results

### Descriptions of the Care Recipients and Their Networks

The 7 networks (NW) that were involved in our research can be distinguished because of varieties between their care situations. Of 4 networks (NW1, NW4, NW6, and NW7), the clients needed care because of physical restrictions, but they were cognitively functioning well and capable of using the DNC app. Their networks consisted mainly of nurses who gave personal care. Informal carers were helping with transportation and arranging tasks and did some household work on an irregular basis. NW1 and NW7 comprised a cohabitated informal carer, a son and a partner, respectively. The client of NW7 had arranged several formal carers via the internet, such as the district nurse who was involved in our research as well. The other 3 networks (NW2, NW3, and NW5) comprised clients who had varying degrees of dementia and were therefore not capable of using the DNC app. They were all fragile, but the client in NW3 was especially restricted in her instrumental daily activities. Further details can be found in Table 2.

### Functional Use of the App

The log-in data we collected over time showed how often communication occurred, who were central senders of e-messages, and who responded to senders. These data gave rise to the insight that the communication varied by the aim for which the app was used (RQ1). The 3 most dominating aims were to (1) arrange care tasks, (2) communicate about the circumstances of the care receiver, and (3) keep in touch/informed about the care situation. Together with characteristics of senders and content of information, 3 different types of online networks surfaced from the data: arranging the care network, discuss the care network, and staying connected network.

#### The “Arranging the Care” Network Type

This type of network is characterized by highly frequent (daily to weekly) usage of the digital agenda, which is used to plan care activities and shifts of caregivers (NW7). The one who directed this digital network is the care recipient himself. His input was focused on making appointments with the formal caregivers. Digital communication occurred most often between the client and the district nurse. The characteristic of the content was pragmatic and concerned the organization of care. The log-in data revealed that the other DNC app members (other nurses, spouse, and relative) logged in regularly for checking information but rarely posted an e-message. During the period of our observation, there was no deterioration in the functional abilities of the client. Therefore, there was no need for additional hours of care over time, which explains the stable usage of the tool in the course of the time. The functional use of the app is clear from the following quote of the care recipient:

...it depends on...is it a privacy issue or not. But if it is a task what can be changed in general, than undoubtedly...it has to be mentioned on the DNC-app, to make everyone aware.

Interview, NW7, client

#### The “Discuss the Care” Network Type

In this type of digital network, the client does not take part himself or herself because of severe cognitive impairments (NW2, NW3, and NW5), and the central informal carer and the district nurse or auxiliary nurse are communicating frequently with each other about the care situation. In NW3 and NW5, there is weekly to monthly activity on the DNC app. The central informal carer of NW2 uses it rarely because of lack of computer skills. In addition, 2 networks (NW2 and NW3) hardly used the digital agenda and action register, but conversely, NW5 used both often. Sometimes the e-messages contained information about housekeeping issues and public health care services concerning the client, such as the following two statements:

As requested by XXX (the auxiliary nurse), I collected bandages from the pharmacy and brought them to mother (they are in the closet where the Care manual is also placed).

Log-in, NW3, informal caregiver

This morning, I paid a visit to your mother. To me, she seemed cheerful. The wound heals well. I am glad that now her bed is at the proper height.

Log-in, NW3, auxiliary nurse

**Table 2:** Characteristics of the care networks at the beginning of the research, number of members of the DNC (digital networked communication) app, and the frequency of using the app during the whole period of observation.

Network	Client, type of helpers, amount and type of help	DNC app users	Frequency of using
1	Middle-aged man, sharing his home with his adult son, average level of education, cognitively functioning well, physically restricted, wheelchair dependent, socially active. Per week, 4 hours of private household work, 3 hours of formal household work, 5 hours of personal care by AN^a^.	1: DN^b^	3 times in the whole period
2	Woman, aged 70 to 75 years, widowed, lives on her own, suffering from dementia, socially active especially with family. Besides her daughter (IC^c^, aged 40 to 45 years), there are 5 family members helping with household work, transportation, and finances. One time a day, AN looks how she is doing. In the mixed care network, both a DN and a CM^d^ are involved.	2: DN, IC	Less than once a month
3	Woman, aged 90 to 95 years, widowed, lives on her own, low level of education, suffering from dementia, restricted in her instrumental daily activities. Besides a son (IC, aged 65 to 70 years), there are 4 other family members helping with household work, transportation, and arranging tasks (3 hours a week). Every day ANs are taking care of heating the food in the microwave, intake of her medication, bathing, and dressing (13 hours a week).	5: DN, IC, AN, private household worker, FM^e^	Once a month
4	Woman, aged 80 to 85 years, widowed, lives on her own, average level of education, cognitively functioning well, physically restricted, wheelchair dependent, socially somewhat restricted. Her IC is an acquaintance (woman, aged 35 to 40 years); she assists the client with arranging tasks. She has a stepdaughter living abroad. Other relatives are a friend and neighbor; they do some household work. ANs come 5 times a day for helping her with meals, personal care, and nursing tasks.	5: CL, DN, IC, AN, FM	Once a week
5	Couple, both suffering from dementia. However, with some difficulty, the woman is able to support her husband a little bit. Aged 80 to 85 years, living on their own, low level of education. Besides a son (IC, aged 40 to 45 years), there are 6 other family members helping with household work, transportation, and arranging tasks. Per week, 3 hours of household work and 3 hours of helping with their medication intake by AN.	6: DN, AN, IC, CM, 2 FMs	Once a week
6	Woman, aged 85 to 90 years, widowed, lives on her own, low level of education, fragile. Besides a daughter (IC, aged 55 to 60 years), there is 1 other daughter helping with household work, transportation, and arranging tasks. Per week, 2 hours household work and 3.5 hours of personal care by AN.	5: CL, DN, IC, AN, FM	Since June more activity
7	Man, aged 60 to 65 years, cohabiting partner. Average level of education, substantial functional restricted, wheelchair dependent. Per week, 32 hours household work, 24 hours personal help, 4 hours nursing tasks, and 2 hours transportation by partner (IC, aged 55 to 60 years). A son, friend, and neighbor help with, for example, transportation and arranging tasks. Per week, 4 hours of household work, 11 hours of personal care, and 7 hours of nursing tasks by DN or/and AN from different agencies.	8: CL^f^, DN, IC, FM, 4 ANs of another agency	More than weekly

^a^AN: auxiliary nurse.

^b^DN: district nurse.

^c^IC: informal carer.

^d^CM: case manager.

^e^FM: family member.

^f^CL: client.

As in the type of network described above, log-in data of this type showed regular log-ins from other helpers just to inform themselves, mostly without leaving an e-message behind. It should be noted that all of the affiliated informal carers of NW3 met each other face-to-face on a regular basis, which reduced the need to actively take part in the DNC. During the study period, the functional abilities of the care recipients decreased, which required adjustment of care activities. This coincided with an increased use of the groupware tool in NW3 and NW5 and digital discussions on specific situations and care needs.

#### The “Staying Connected” Network Type

Differing from the other 2 network types, this network type comprised an equal contribution in communication by the client, the informal carer, and the nurse (NW4 and NW6). During the entire period of observation, the log-in data showed a low level of activity. Compared with the *discuss the care network*, the necessity for DNC seemed to be missing because the care recipients were still able to communicate themselves, and there was no deterioration in their functional abilities and no change in care needs in the period of observation. Therefore, quite a lot of e-messages were not about care but about leisure spending of the care recipients, periods of absence of the carers, and daily issues. Both care recipients explained that they experienced a lack of benefit of using the tool. They preferred communicating by phone or face-to-face to digital communication:

If I want to go out this afternoon, I can put a message on the app, but she (the auxiliary nurse) might not see it on time. That’s why I prefer to call her by phone.

Interview, NW4, client

The central informal carers of NW4 and NW6 articulated that the possibility to log in on to the DNC app increased the peace of mind. Although not living nearby, in a split second, the carers can be alarmed by receiving a push message if the health condition deteriorates. Otherwise, if everything is going well with their beloved one, they were reassured by reading e-messages such as this one:

How kind of you sending me a message! I’m doing well, hope you’re fine. Dear regards

Log-in, NW6, client

### Consequences of Using the Digital Communication App

During the interviews, we asked clients and their helpers what might be the effects of using the DNC app for communication on care, the efforts from informal carers to help, and the perceived quality of care (RQ2). The forms that were supplied to the clients and informal carers included relevant questions, with scales to measure changes herein during the period of observation.

#### Effects on Communication on Care

In general, the participants seemed to agree with each other that using the DNC app contributes to a quicker, better, and intensified connection between the affiliated members of the mixed home care network. Especially, the informal carers emphasized the advantage that everyone can be immediately aware of what is going on. By logging in, the information became visible, so everyone can be informed about which task has to be done by whom:

Well, you see...for example...look here, here are the appointments [He shows the interface on his laptop]. This is an enumeration of work to do, we arranged that by mutual discussion. My brother, weekly he buys the groceries. My girlfriend and I are the back up. My other brother does the garden, and his sons are the back up.

Interview, NW5, informal carer

Despite their low use of the tool, the informal carers in the *staying connected* network type appreciated the opportunity to see online information that would have been otherwise obtained by reading the hard-copy dossier at the client’s home. One of the informal carers, therefore, stressed that the ability to have short lines in the triangle of client-caregiver-professionals was the main motivator for her to use the DNC app:

In the past week I have checked the app and it decreases the necessity for other modes of contact. We will use it more to maintain the communication within the triangle.

Interview, NW3, informal caregiver

In contrast, the client of NW7 mentioned several shortcomings of the tool that hindered coordination of caring tasks. Most of them were functional issues such as the inability to rehearse arrangements in the agenda in a simple way. Notably, the clients of NW4 and NW6 expressed that the DNC app would be an innovative idea for carers of people with dementia but not for themselves because they are cognitively still functioning well. Therefore, they prefer face-to-face or phone consultation rather than digital contact to coordinate care. Finally, Table 2 shows that during the period of observation, there was no mutual communication at all in NW1. The reason to not use the DNC app was their familiarity with other digital communication tools. Besides that, the helping family members lived nearby, so there was no need for them as well. Overall, for all the members of NW1, there were not enough triggers to use the groupware tool. However, till the end of the project, they were eager to provide information on effects of using mainly WhatsApp to coordinate all types of helping tasks. One of the consequences was that except for arranging transportation, they hardly communicated by phone anymore.

For the home care workers, the downside of the DNC app was the huge amount of posts to be read by them. A lot of the nurses read the e-messages in their spare time, especially the ones who had not yet received an appropriate mobile phone from the agency. Furthermore, during the period of observation, there was the disadvantage of the existence of various methods to submit the same information, such as via the DNC app, on the intranet of the care agency and in the dossier at client’s home.

#### Effects on Degree of Help From Informal Carers

At the end of our period of observation of only 6 months, it was not possible to determine whether the e-tool had contributed to a statistically significant increase of informal help within the networks. The sample was too small to calculate differences. Neither was it possible to establish a significant decrease of formal help. If more helping hours per week were reported at T2 compared with T0, it seemed related to an increased demand for care of the client. However, by interviewing the informal carers 3 consecutive times, we can conclude that they became more engaged with the tool and that they felt more comfortable using the app later than in the beginning.

It is relevant to mention here that some home care workers expressed that they gained more insight into the degree of resilience of informal carers:

Although we see on the DNC-app only elaborations about care situations, it’s ok. It gives us a chance to determine their ability to care.

Interview, district nurse NW1-5

Moreover, not earlier than in the final phase of the study, we saw in the log-in data some cautious insinuations from nurses to get a task done by an informal carer:

Would/could you discuss with the general practitioner which medical options there are to lessen the pain of your mother? Please, take into account that some medication increases the risk of falling.

Log-in, NW3, district nurse

On the other hand, one of the district nurses said she felt encumbered to ask for more help if only 1 informal carer is connected on the DNC app.

#### Effects on Quality of Experienced Care

Noticeably, the client who showed the highest degree of acceptance to use the groupware tool was the frailest of our research group. Due to his physical limitations and the large number of caregivers, he was very enthusiastic about using the app:

The app is the central point where carers can find the latest information. It gives me confidence that they know what to expect and how to handle.

Interview, NW7, client

His central formal carer confirmed this improvement in quality from her perspective:

The DNC-app increases the client’s self-efficacy on his life and caring tasks.

Interview, NW7, district nurse

The formal caregivers mostly admitted that the DNC app could be helpful when dealing with complex care and that the tool could also relieve their daily work practice:

Before we go to the house of the client we can inform and prepare ourselves just by logging in on the DNC-app.

Group session, auxiliary nurse

Finally, to enhance the quality of care, some affiliated members suggested to invite other disciplines besides the participants of the mixed care network. For example, the general practitioner, dementia case manager, and physiotherapist could be hooked onto the DNC app as well. In this case, some nurses expressed, for example, that photos that provide insight into the healing tendency of wounds can be exchanged. At the same time, this elicited hesitations as photos and medical information provoke issues such as privacy and integrity.

### Organizational Conditions Which Facilitate or Restrict the Implementation of the Digital Networked Communication App

The data collected from home care workers and their managers showed that different factors contributed to (non)use of the DNC app (RQ3). The coordination of care was mentioned several times by the managers as an important potential benefit of the app but in the first place, they perceived this type of software to be a tool to monitor the care situation of their frail clients. Monitoring is necessary for safety reasons and for getting information about what can be done by the informal carers and when and how formal care is needed. However, the management also stressed to be cautious because the use of the DNC app is limited, given the data protection legislation.

Regarding the existing workflow of the home care workers, the recent reforms in long-term care are mentioned by auxiliary nurses several times as a barrier to implement the app. Due to a decrease in time for care provision, they see lesser opportunities to use the tool. Although their pessimism reduced in the course of time, during the T1 meeting, they were told to be distrustful because the DNC app would reduce their leisure time. They feared receiving a notification on their mobile phone after working hours, which needed to be taken care off. In addition, an auxiliary nurse expected that the app would lower the threshold for informal carers to ask nurses doing tasks that did not belong to their responsibilities (anymore). In contrast, the district nurses showed more enthusiasm from the start in adopting tools such as the DNC app. In a later stage of the implementation, the attitude toward the app of both types of nurses became more similar. They saw as potential benefit that links between the informal and formal carers can be shortened by the app if appropriate agreements are made:

If you have questions about their father or mother, and they just do not pick up the phone, then it may take too long before we have connection with each other. When they decide to use the app to communicate with us, then there is a commitment. In that case, they should at least once a week have to deal with the DNC-app.

Focus group session, team manager

The use of the app can be hampered when it is not clear who is responsible for assisting the informal carers in the use of the device. The following 2 statements make it clear that an informal carer had difficulties with the DNC app but was motivated to get instructions from the app developer:

...and I had as well difficulties with another functionality. I tried a few times, without succeeding. Yeah, what to do about it.

Interview, NW2, informal carer

The developer comes this week to help me, I hope he don’t forget it. We have to wait again.

Log-in, NW2, informal carer

The district nurse of NW2 is, during the T2 interview, clear in her opinion that the developer failed to take his responsibility to instruct this client in using the DNC app:

I thought he is the one who should have helped her till she knows how to use it.

Interview, NW2, district nurse

## Discussion

### Principal Findings

More reliance on informal caregivers of older people living at home asks for more connection and communication among all types of caregivers. Therefore, the main aim of this study was to explore the variety of DNC within different types of mixed home care networks. Studying the qualitative and quantitative information gave us insight into which aspects the 3 types of actors (clients, informal, and formal carers) showed similarities and differences in views on using a DNC app to communicate with each other and barriers and facilitators for its implementation.

Overall, such a digital tool in a closed network can facilitate communication on care between the client and informal and formal carers. How it will be used depends largely on the involvement of the client. If the client has a high capacity to use the tool and engagement to optimize the coordination, it may lead to more use of the agenda instead of communication about care. On the other hand, in case of lower involvement or absence of involvement of the client, the likelihood of communication between carers increases, especially when the informal helper has a strong motivation to use the digital tool. In small offline networks when the care situation is stable and the client has no or little involvement, a digital network surfaces, which exists but is not used very often. In that case, the informal carer uses the tool for his peace of mind. Looking at these insights, there is a tendency to say that the sources and content of communication of online networks, in particular, reflect the needs, capabilities, and attitudes toward DNC of the care recipient and the informal caregivers, and to a lesser degree, the structure of the care network present.

However, the e-tool seems to be of particular use to mixed care networks with many different caregivers. In this type of network, it is not necessary that all participants are communicating online with each other, as care can be arranged among just a few of them; however, their e-messages are available for everybody when they log in. Therefore, the most important benefits of the tool for the client and his carers seem to be the easy availability of up-to-date information, the ability to give a sense of safety for the carers, and short communication lines in case of complex care situations. On the other hand, there is no clarity yet about whether electronic communication contributes to an increase of hours and types of informal help or to quality of life of clients and caregivers.

### Remarks and Recommendations

On the basis of our findings, several steps can be identified that home care organizations need to take when starting to use a groupware tool. The first step is to identify the targets that the use of the app may deliver. One such target could be to work more efficiently as face-to-face contact with affiliated members is replaced by online contact, which saves time. Other targets may be a larger involvement of informal carers and improved coordination of care within the mixed home care network. Second, the home care workers need to select clients and their caregivers for whom the groupware tool might work. This, in particular, concerns capable clients and caregivers who value digital communication [12]. The third step is to identify the mixed home care network around the determined client and arrange a meeting about who to invite to connect on the groupware tool, what type of communication is most appropriate, and how often communication is expected. The 3 types of digital care networks identified in this study can be used as examples for the home care staff in which different agreements with clients and caregivers can be made. Finally, continuous monitoring of the actual use of the app is warranted. Care situations change, which need to be reflected in the use of the app. For example, using only the calendar can be sufficient when the client is cognitively functioning well but may fall short as his or her health deteriorates. In that case, adjustments in the way of communicating are necessary because the digital *arranging the care* network may need to become a *discuss the care* network type.

### Strengths and Limitations of the Study

The strengths of our design are that multiple types of participants (clients, caregivers, and managers) were interviewed, thereby representing a wide range of perspectives. Furthermore, multiple ways of data collection made it possible to come up with rich information to answer our research questions and to contribute to the process of theorizing new sociological phenomena as DNC.

There are also limitations of the study. First, there were few specific inclusion criteria for the pilot study, so there is a high variability among the cases included. Second, the small size of the sample and the low use of the tool limited statistical analyses to corroborate our findings. Moreover, the period of observation was relatively short and did not allow for significant changes in structural and functional characteristics of the mixed care networks. It can be concluded that longitudinal large-scale studies are needed to examine how a tool such as the DNC app can indeed affect communication among caregivers.

### Conclusions

This small-scale study is one of the first to report on digital communication tools in mixed home care networks. Due to the information collected with multiple methods and from different types of actors, we were able to come up with (RQ1) 3 types of communication patterns in home care networks to illustrate the different functional uses of the groupware tool. These differences are clearly related to the physical frailty status of the care recipient, the motivation of client and informal caregivers, and the opportunities for the formal caregivers to use the tool and are less related to the structural features of the care network. Although the online care network may be rather comparable with the offline care network, digital communication is limited to specific network members and mostly focused on the arrangement of care in times that this was most needed. Those characteristics (RQ2) enhanced the care management of the digitally literate care recipients, the feelings of safety among informal caregivers, and efficiency of organization by the formal caregivers, which are all basic ingredients of good quality of care [24]. Before actually using an e-tool, it is important to (RQ3) consider the several barriers and facilitators of success when implementing it into home care organizations. For example, groupware tools have less privacy problems than eHealth and care records, which may be a trigger to implement the tools in home care organizations [25]. Finally, DNC may enhance the linkages in the triangle of client, informal caregiver, and professional caregiver and increase peace of mind among all users.

## Abbreviations

AN: auxiliary nurse
CM: case manager
CL: client
DN: district nurse
DNC: digital networked communication
eHealth: electronic health
e-message: electronic message
e-tool: electronic tool
FM: family member
IC: informal carer
NW: network
RQ: research question
